# Cellulose-based magnetoelectric composites

**DOI:** 10.1038/s41467-017-00034-4

**Published:** 2017-06-28

**Authors:** Yan Zong, Tian Zheng, Pedro Martins, S. Lanceros-Mendez, Zhilian Yue, Michael J. Higgins

**Affiliations:** 1 ARC Centre for Electromaterials Science (ACES), Intelligent Polymer Research Institute/AIIM Faculty, Innovation Campus, Squires Way, University of Wollongong, Wollongong,, NSW 2522 Australia; 2 Centro/Departamento de Física, Universidade do Minho, Braga, 4710-057 Portugal; 3 BCMaterials, Basque Center for Materials, Applications and Nanostructures, Parque Tecnologico de Bizkaia, Derio, 48160 Spain; 4 IKERBASQUE, Basque Foundation for Science, Bilbao, 48013 Spain

## Abstract

Since the first magnetoelectric polymer composites were fabricated more than a decade ago, there has been a reluctance to use piezoelectric polymers other than poly(vinylidene fluoride) and its copolymers due to their well-defined piezoelectric mechanism and high piezoelectric coefficients that lead to superior magnetoelectric coefficients of >1 V cm^−1^ Oe^−1^. This is the current situation despite the potential for other piezoelectric polymers, such as natural biopolymers, to bring unique, added-value properties and functions to magnetoelectric composite devices. Here we demonstrate a cellulose-based magnetoelectric laminate composite that produces considerable magnetoelectric coefficients of ≈1.5 V cm^−1^ Oe^−1^, comprising a Fano resonance that is ubiquitous in the field of physics, such as photonics, though never experimentally observed in magnetoelectric composites. The work successfully demonstrates the concept of exploring new advances in using biopolymers in magnetoelectric composites, particularly cellulose, which is increasingly employed as a renewable, low-cost, easily processable and degradable material.

## Abstract (web summary)

Magnetoelectric materials by converting a magnetic input to a voltage output holds promise in contactless electrodes that find applications from energy harvesting to sensing. Zong et al. report a promising laminate composite that combines a piezoelectric biopolymer, cellulose, and a magnetic material.

## Introduction

Magnetoelectric (ME) materials undergo dielectric (magnetic) polarization in an external magnetic (electric) field^1, 2^. Compared to rare single phase ME crystals (e.g., Cr_2_O_3_)^3^, composites with ME properties are attractive for their ease of processing and, importantly, superior ME response at room temperature^4, 5^ to enable practical devices such as sensors for ultralow magnetic field detection^6^. ME composites consist of both piezoelectric and magnetostrictive components, and the ME effect of the composite is not the natural property per se, but is actually the result of tensor properties^7^. That is, when an external magnetic field is applied to the composite, the magnetic component changes its shape magnetostrictively to induce strain on the piezoelectric component, causing dielectric polarization. This uniquely indirect two-phase strain coupling provides the flexibility of optimizing both the piezoelectric phase, magnetostrictive phase, and their interface to enhance the ME response^8^.

**Fig. 1:**
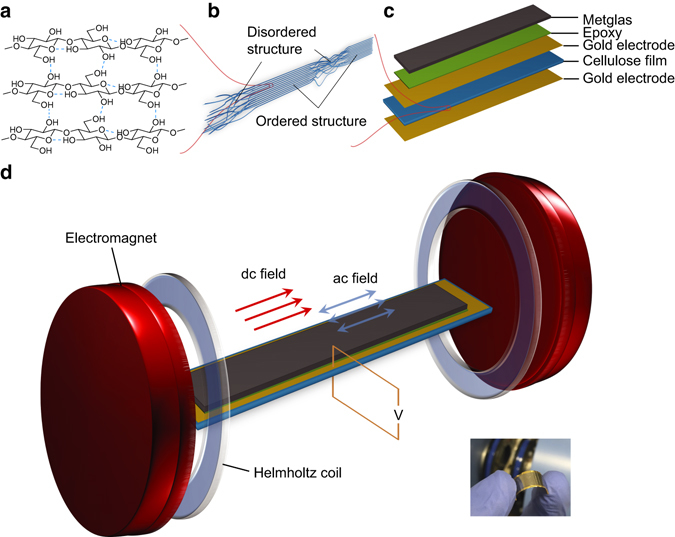
Cellulose ME laminate and the experimental set-up of ME effect measurement. **a** Scheme of cellulose crystal II, the most common crystalline type in regenerated cellulose materials. The saccharide unit provides dipolar segments along the aligned fibril thus rendering the piezoelectric nature. **b** Illustration of cellulose fibril alignment at the cross-section of cellulose film. Part of the ordered structure provides crystalline properties. **c** Schematic of cellulose-based ME laminate structure. The thickness is measured as 19 ± 2 µm for hot-press film and 27 ± 3 µm for the control film by using a micrometer caliper. The cellulose films are first sputter coated with 50 nm thick gold electrodes on both sides and then glued with Metglas 2605 SA1, of which the thickness is 25 µm. To ensure even distribution, the epoxy is preheated to 60 °C to improve the liquidity. **d** Schematic view of the bulk system for ME voltage measurement. The output voltage is collected from the interface gold electrodes and monitored as root mean square values using a lock-in amplifier. The inset below shows the picture of the final cellulose ME laminate used in the measurements

Generally, the use of magnetostrictive materials with high magnetic permeability and low field saturation, such as Metglas^9, 10^, provides the possibility of inducing fast mechanical deformation under relatively weak magnetic fields. Combining Metglas with the highest piezoresponsive polymers, such as poly(vinylidene fluoride) (PVDF)^11^, gives rise to significant strain transfer and the highest ME voltage outputs. As such, PVDF and its copolymers have been exclusively studied since the first ME polymer composite consisting of PVDF was demonstrated in 2002 and expected developments in exploring other types of piezoelectric polymers have not been forthcoming. This has led to the emergence of a central dogma where PVDF is viewed as the “material of merit”, despite the enormous potential for other polymers to bring significant added-value properties and function to ME composite devices. Actually in nature, there are numerous biomolecules possessing piezoelectricity and the practical applications arising from them has attracted a lot of research interest^12, 13^. Exploring advances in harnessing the unique structures and properties of naturally occurring piezoelectric biopolymers will transpose the aforementioned central dogma and broaden the application base for ME composites. In particular, cellulose is the most substantial organic substance in nature^14^ and the origin of its piezoelectric response, discovered early in wood^15^, comes from inherent crystallinity^16^ (Fig. 1a). As a very cheap and renewable material, it is developing rapidly as a supporting substrate for flexible and transparent electronics^17, 18^, and recently fabricated as piezoelectric paper for actuation, energy harvesting, and acoustic applications^19^.

Here we first demonstrate the fabrication of ME composites based on the natural biopolymer, cellulose. In regenerated cellulose, the degree of crystallinity is usually at a low level and improving the alignment of cellulose fibrils, e.g., either through stretching or heat, is an effective method to enhance piezoelectricity, as the ordered structure (Fig. 1b) is preferred for crystalline generation^19, 20^. To fabricate the cellulose-based ME composites, we prepare laminate (bilayer) structures comprising Metglas and cellulose films, enabling two-phase strain coupling for an enhanced ME response, that is superior to previously reported composite structures^21^. Cellulose films are obtained from regular solution processing methods and the pristine wet film is aired at room temperature or alternatively hot pressed (60 °C) to assess the effect of heat treatment on the cellulose crystallinity and overall ME response (see ‘Methods’ section). ME laminates are assembled by combining a cellulose film, consisting of gold layers sputtered on both sides as inter-face electrodes, with a Metglas (the magnetostrictive phase) film by gluing the two components together using Devcon epoxy to enable strain coupling (Fig. 1c). The final laminate composite is tested using a dynamic method, involving the application of an alternating (**H**_ac_) magnetic field superimposed on a constant (**H**_dc_) magnetic field, to quantify the ME frequency response and output voltage (Fig. 1d).

## Results

### ME properties of cellulose/Metglas laminate composites

**Fig. 2:**
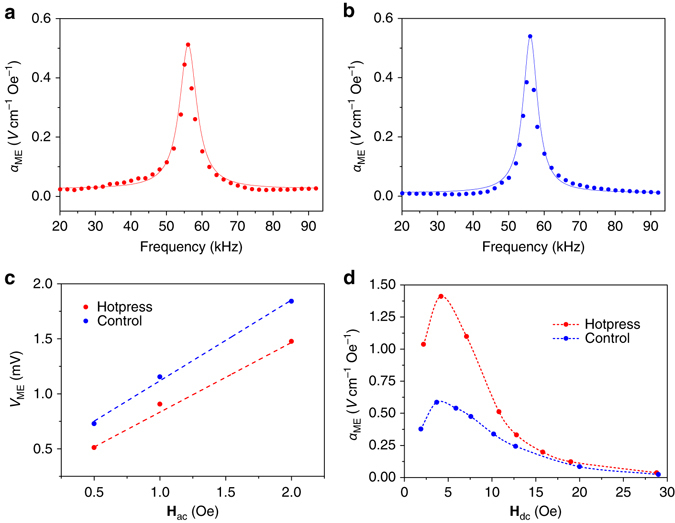
ME effect of cellulose-Metglas laminates. **a**, **b** ME voltage coefficient as a function of **H**_ac_ frequency when **a H**_dc_ = 10.8 Oe for hot-press sample and **b H**_dc_ = 5.9 Oe for the control sample. The experimental data points are fitted to a Lorentzian resonance model (the *solid line*) and the resonance peaks found at 56.1 kHz. **c** The resonant ME output voltage as a function of applied **H**_dc_ strength. The ME laminates were induced by **H**_dc_ = 10.8 Oe for hot-press sample and **H**_dc_ = 5.9 Oe for the control sample at which a Lorentzian resonance profile has been observed. **d** Resonance enhanced *α*_ME_ as a function of **H**_dc_ for hot press (*red dotted line*) and control (*blue dotted line*). All data are obtained under **H**_ac_ = 0.5 Oe

The ME voltage coefficient (*α*_ME_) is an important parameter that evaluates the ME effect of a material, and is defined as:

1 $${\alpha _{{\rm{ME}}}}{\rm{ = }}\frac{{{\rm{d}}{\bf{E}}}}{{{\rm{d}}{\bf{H}}}}$$

where **E** and **H** represent the strength of the electrical and magnetic fields, respectively. *α*_ME_ is calculated as the ME output voltage (V) per unit of cellulose film thickness (cm) and **H**_ac_ strength (Oe). To measure the ME response, we first fix the **H**_dc_ and **H**_ac_ strength and *α*_ME_ is recorded as the function of the **H**_ac_ frequency. For both the air-dried and hot-pressed cellulose ME laminates, a significant increase in *α*_ME_ is observed with a peak maximum at **H**_ac_ frequency of ~56.1 kHz (Fig. 2a, b), indicating a resonance enhancement effect that is characteristic of an ME response in laminate structures^21^. In this case, once the **H**_**ac**_ frequency coincides with the resonant frequency of the magnetostrictive structure (i.e., Metglas film), the mechanical strain is amplified, thus causing a significant enhancement in the *α*_ME_. The resonant frequency (*f*_r_) of the magnetostrictive layer, if oscillating with a free end, depends on its density (*ρ*), Young’s modulus (*E*), and the length (*L*) along the magnetic field^22^, and is given by:

2 $${f_{\boldsymbol{\rm r}}} = \frac{1}{{2L}} \times \sqrt {E{\rm{/}}\rho } $$

**Fig. 3:**
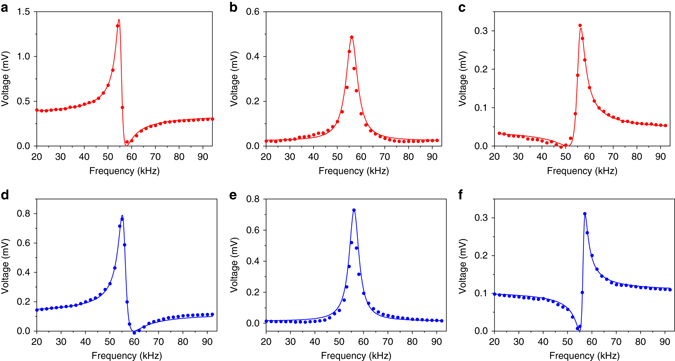
Representation of anti-resonance effect of ME output voltage. **a**–**c** ME output voltage of hot-press sample as a function of **H**_ac_ frequency under **a H**_dc_ = 4.2 Oe, **b H**_dc_ = 10.8 Oe, and **c H**_dc_ = 12.8 Oe. **d**–**f** ME output voltage of air-dried sample as a function of **H**_ac_ frequency under **d H**_dc_ = 3.9 Oe, **e H**_dc_ = 5.9 Oe, and **f H**_dc_ = 12.7 Oe. The experimental data (*dots*) are fitted to a modified Maxwell Eq. 4 or Lorentzian function (shown as *solid lines*)

For Metglas, *E* = 100–110 GPa and *ρ* = 7.18 × 10^3^ kg m^−3^. In our case *L* = 36 mm, thus the calculated theoretical *f*_r_ of 51.8–54.4 kHz is very close to the experimental *α*_ME_ resonance peak values in Fig. 2a, b. For Fig. 2a, b, ME responses are specifically shown for different applied **H**_dc_ of 10.8 Oe and 5.9 Oe for the hot-pressed and air-dried samples, respectively, because these **H**_dc_ produce a symmetrical Lorentzian resonance profile as opposed to a non-symmetrical profile, which is discussed later in Fig. 3. Displayed in Fig. 2c is the actual resonant output voltage (*V*_ME_) under different **H**_ac_ strength while the **H**_dc_ strength is fixed at the respective values given in Fig. 2a, b. Under these **H**_dc_ strengths, the *V*_ME_ of the air dried is slightly higher than the hot pressed, and the linear increase in both as a function of **H**_ac_ further confirms the existence of an ME effect (Fig. 2c). Despite the air-dried samples having higher *V*_ME_ for the symmetrical Lorentzian resonance condition, the hot-pressed samples gave significantly higher *α*_ME_ at all other **H**_dc_ strengths (Fig. 2d). More specifically, Fig. 2d shows the resonance *α*_ME_ as a function of **H**_dc_ strength. The hot-press samples show a maximum *α*_ME_ at **H**_dc_ = 4.2 Oe, as high as 1.41 V cm^−1^ Oe^−1^, which is importantly considered to be in the practically useful range^11^. Comparatively, the air-dried sample shows a similar **H**_dc_-dependent behavior, with significantly less maximum *α*_ME_ of 0.59 V cm^−1^ Oe^−1^ occurring at **H**_dc_ = 3.9 Oe. Furthermore, it is expected that the maximum ME output voltage can be further enhanced by increasing **H**_ac_, as given by the linear relationship (i.e., 1.48 mV for the hot-pressed sample when **H**_ac_ = 2.0 Oe) in Fig. 2c ^23, 24^.

The *α*_ME_ is also derived from a ME equivalent circuit method^25^, which is expressed as^9^:

3 $${\alpha _{{\rm{ME}}}} = \frac{{n{d_{33,{\rm m}}}{d_{31,{\rm p}}}}}{{n{\varepsilon _0}\varepsilon _{33}^SS_{11}^E{\rm{ + }}\left( {1 - n} \right)S_{33}^H\left( {{\varepsilon _0}\varepsilon _{33}^S{\rm{ + }}d_{31,p}^2{\rm{/}}s_{11}^E} \right)}}$$

where *n* is the magnetic phase thickness ratio $$s_{11}^{E} $$, and $$\varepsilon _{33}^S$$ are the elastic compliances of the piezoelectric and magnetostrictive layers. *ε*_0_ and $$\varepsilon _{33}^S$$ are the vacuum permittivity and the dielectric constant of piezoelectric layer at constant strain. *d*_33,m_ and *d*_31,p_ are the longitudinal piezomagnetic and transverse piezoelectric coefficients, respectively. According to the measured *α*_ME_, elastic moduli (Supplementary Fig. 1)^26^ and dielectric constant^27^ of regenerated cellulose films, and the magnetostrictive coefficient of Metglas SA1^9^, the effective *d*_31,p_ is estimated as 5.95 and 1.55 pC N^−1^ for hot-press and air-dried cellulose films, respectively. These values are similar to early reported results of pristine and electrically aligned cellulose films^28^ but is much lower than those of optimized PVDF copolymers (the highest *d*_31_ can reach up to 28.6 pC N^−1^), and by using this polymer coupling with Metglas SA1, the maximum *α*_ME_ of 320 V cm^−1^ Oe^−1^ has been achieved^29^. Since the Young’s modulus are measured as 3.95 GPa for both the two cellulose films, the ME response should mainly depend on the crystallinity degree of the regenerated cellulose. Thus, it is reasonable to assume that optimization of cellulose matrix could be an effective way to enhance the piezoelectric effect and further improve the *α*_ME_ of the cellulose-based ME composite.

### Fano resonance of the ME effect

Remarkably, a Fano-resonance effect is observed to accompany the resonance enhancement (Fig. 3). First, the hot press shows a symmetrical Lorentzian resonance profile at an applied **H**_dc_ = 10.8 Oe (Fig. 3b) and as given earlier in Fig. 2a. However, a Fano-like resonance, featuring an anti-resonance peak, appears when the **H**_dc_ is below (Fig. 3a) and above (Fig. 3c) the **H**_dc_ of 10.8 Oe. At a **H**_dc_ < 10.8 Oe, the anti-resonance peak occurs at a frequency value that is higher than the resonant peak (Fig. 3a), while if **H**_dc_ > 10.8 Oe, the anti-resonance damping peak occurs at lower frequencies (Fig. 3c). Similarly, the air-dried sample shows a Fano-like resonance, with a shift in the anti-resonance occurring at **H**_dc_ above and below the **H**_dc_ (5.9 Oe) for the symmetrical Lorentzian resonance (Fig. 3d–f).

To assess the Fano-resonance profile, the *α*_ME_ output voltage curves measured as a function of **H**_dc_ are fitted to a modified Maxwell equation:

4 $$V\left( \omega \right) \, {\rm{ = }} \, A\left| {\frac{{{\omega ^2} - 2i{\delta _{\rm{a}}}{\omega _{\rm{a}}} {\omega - }\omega _{\rm{a}}^2}}{{{\omega ^2} - 2i{\delta _{\rm{r}}}{\omega _{\rm{r}}} {\omega - } \omega _{\rm{r}}^2}}} \right|{\rm{ + }}a\omega {\rm{ + }}b$$

where *A* is the amplitude constant. *ω*_r_ = *2πf*_r_ is the resonance frequency and *ω*_a_ = *2πf*_a_ is the anti-resonance frequency. *δ*_r_ and *δ*_a_ are the damping constants for the resonance and anti-resonance, respectively. *a* is the constant corresponding to a linear background voltage and *b* is the factor fitting the imaginary section to experimental data. We subtracted the background noise and only present the actual ME output voltage here. Fits to the data (solid line) are given in Fig. 3 and all fitting parameters are summarized for the hot-pressed and air-dried samples in Supplementary Tables 1 and 2.

Fano resonances are widely observed in different fields of physics, e.g., photonic materials and crystals^30^, and recently reviewed for plasmonic nanoparticles and metamaterials^31^. They are also seen in electromechanical coupling of piezoelectric materials, e.g., in impedance measurements of common lead zirconate titanate^32^ and generally described as occurring due the interference between continuum and discrete states. However, the Fano resonances of piezoelectric materials have shown not to impose on the frequency line shape of ME responses^32^ and therefore until now have not yet been experimentally observed in ME composites, including both polymer (i.e., PVDF) and surprisingly ceramic-based composites that were first established more than four decades ago. Theoretical modeling has predicted Fano resonances either as a conductivity change^33^ or the interference of two excitation pathways of ME laminate cantilever structures^34^, or those oscillating with free ends^35^. Though despite these models, the lack of experimental evidence suggests that cellulose as a piezoelectric material within ME composites possess unique piezoelectric mechanisms, mechanical properties, and coupling that contribute to the occurrence of a Fano resonance.

To further understand the origin of the Fano resonance, the possible role that water may play is investigated by assessing cellulose with varying degrees of water content. Compared to the ME frequency response of cellulose films prepared at room temperature and with hot pressing under 60 °C (Figs. 2 and 3), cellulose films hot pressed at higher temperatures of 100 °C to remove most of the residual water also shows a Fano-resonance effect with the frequency peak of the symmetric Lorentz profile occurring at significantly higher **H**_dc_ field (Supplementary Fig. 2). More specifically, the resonance peak shifts from 5.9 Oe in air-dried sample and 10.8 Oe in the 60 °C hot-pressed samples up to 13.1 Oe in the 100 °C hot-pressed sample (Supplementary Fig. 3a). Shifts in the frequency values of the anti-resonance, either positioned above or below the resonance peak, also occur due to variations in the water content (Supplementary Fig. 3b) and are qualitatively related to changes in the quality factor, i.e., damping of the ME frequency response. That is, with less water content the resonance and anti-resonance peaks become broader (Supplementary Fig. 2, hot pressed 100 °C) as opposed to being narrower if the water content is increased (Fig. 3a–c, hot pressed 60 °C). A further increase in water content gives the narrowest resonance peak, or highest-quality factor, in the air-dried films (Fig. 3a–c, air dried). These changes in the frequency profile indicate that water has a significant effect on the ME response, including the Fano resonance, which still persists when removal of most water is expected. In an attempt to completely eliminate the effects of water and to produce highly homogenous crystalline cellulose, without the amorphous regions found in regenerated cellulose, we prepare ME laminate composites based on nanocrystalline cellulose to further elucidate the Fano resonance. In this case, the highly crystalline form has almost no porous structure, thus minimizing the effect of residual water and heterogeneity (i.e., crystalline vs. amorphous regions) in the piezoelectric layer. From these ME composites using the same configuration and dimensions, a Fano resonance is present although the anti-resonance only occurs at frequencies below the resonance and extraordinarily no symmetrical resonance profile is observed at any applied **H**_dc_ field (Supplementary Fig. 4). Collectively, water plays an ostensible role in the ME response; however, experiments using nanocrystalline cellulose suggest that it is the unique and inherent chemical structure of cellulose that gives rise to the Fano-resonance effect.

At the bulk scale, the strain coupling of the piezoelectric and magnetostrictive can be divided into two pathways: the tension strain caused by magnetostriction and bending strain that is attributed to the configurational asymmetry of a bilayer, shrinkage of epoxy while drying, and mechanical properties of the cellulose. When the tension and bending strains are at equilibrium, the resonance frequency curve of the *α*_ME_ output voltage is a symmetrical Lorentzian profile. However, at **H**_dc_ values less or greater than **H**_dc_ = equilibrium, where the driving force for tension strain is either too weak or strong, then interference coupling on the equilibrium bending-tension strain results in a Fano resonance profile.

The equilibrium strain coupling giving a symmetrical Lorentzian profile is observed at **H**_dc_ = 10.8 Oe for the hot pressed (Fig. 2a) and **H**_dc_ = 5.9 Oe for air dried (Fig. 2b). Since equivalent procedures and magnetostrictive components are used to fabricate the laminates, a difference in the anti-resonance dependence on **H**_dc_, as well as the damping coefficients (Supplementary Tables 1 and 2), from the fitting are likely to be attributed to the different frequency-dependent strain transfer and tensile capacity of the cellulose as a consequence of their different processing methods (i.e., air dried vs. hot pressed). Further to this, anti-resonance is useful for deconvolving the properties of complex mechanically coupled systems, and will be fundamentally important for elucidating the ME mechanisms in cellulose.

### Crystallinity and morphology of cellulose

**Fig. 4:**
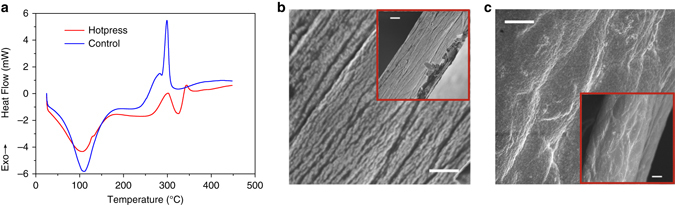
The effect of treatment on cellulose crystallinity and morphology. **a** DSC thermograms of hot-press (*red line*) and control (*blue line*) films. The melting and oxidation temperatures are 302.9 and 345.5 °C for hot-press film; 281.3 and 299.0 °C for air-dried film. The endothermic peak in this range corresponds to melting process of cellulose^42^. The difference of oxidation (decomposition) temperature of the two films has been verified by using TGA (Supplementary Fig. 5). **b**, **c** SEM cross-section images of **b** hot-pressed and **c** air-dried cellulose films. The magnification is ×15,000 for detailed view (scale bar, 1 µm) and ×5,000 for full view (inset, scale bar, 2 µm)

The overall increased performance of hot-press cellulose ME laminate compared to the air dried (Fig. 2d) is presumably due to the piezoelectric properties of each cellulose film, as determined by the extent of crystallinity. To verify this, differential scanning calorimetry (DSC) is employed to study hot-pressed and air-dried cellulose films by quantifying the heat associated with melting of the polymer (Fig. 4a). Thermal analysis of the endothermic peaks corresponding to the melting process indicates the hot-pressed sample adsorbs significantly higher energy, with fusion enthalpies of 50.49 J g^−1^ and 9.39 J g^−1^ for the hot pressed and air dried, respectively, suggesting a higher crystalline content in former. Further, thermogravimetric analyses indicate that the two different cellulose decompose at different temperature (Supplementary Fig. 4). The difference of crystalline content is explained by changes in cellulose morphology, as evident in scanning electron microscopy (SEM) images of film cross-sections (Fig. 4b, c). In hot-pressed films, the cellulose displays a layered, or aligned fibrous structure, consisting of smaller fibrils with uniform distribution along the longitudinal direction of the film (Fig. 4b). In contrast, the air dried shows a less distinct anisotropic structure, particularly with the absence of smaller fibril structures (Fig. 4c).

### Piezoelectric properties of cellulose by piezoresponse force microscopy

**Fig. 5:**
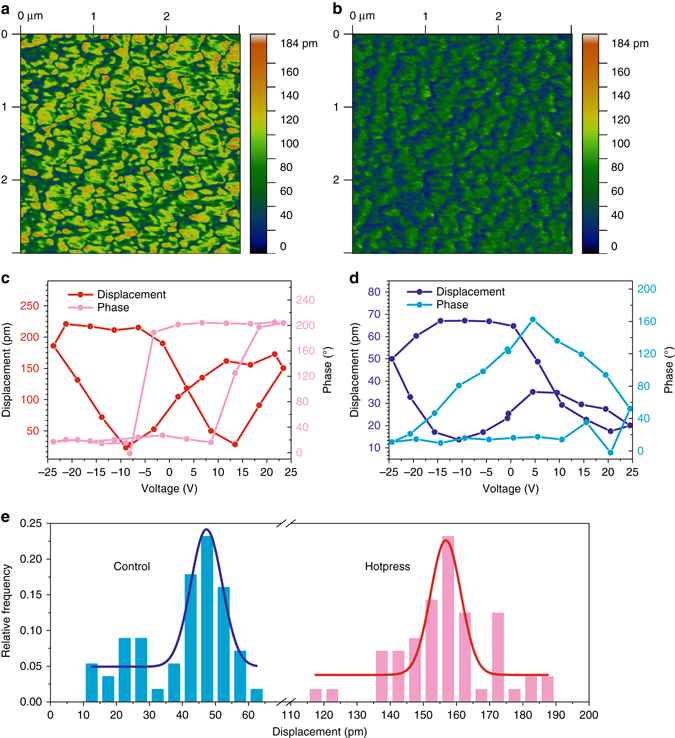
Effect of treatment on piezoelectric response measured by PFM. **a**, **b** PFM amplitude images of **a** hot-press and **b** air-dried samples. A conductive tip is used to apply a constant bias of 9.4 V to induce local ME displacement while imaging. **c**, **d** Piezoelectric butterfly loops of **c** hot-press and **d** control cellulose films elucidated by using SS-PFM. The *red* and *blue dots* are the hysteresis loops representing the bias-induce amplitude displacement. The *pink* and *cyan dots* represent phase changes corresponding to the hysteresis loops. **e** Histograms of bias induced amplitude displacement at applied voltage of −25 V during SS-PFM measurements

Piezoresponse force microscopy (PFM) is used to understand the relationship between the cellulose crystallinity, residual water content, ME output voltage, and the local piezoelectric properties of the hot-press vs. air-dried films. Corresponding PFM height images reveal topography does not significantly differ (Supplementary Fig. 6), while the spatial distribution of the piezoelectric nanoscale domains are similar for the hot-pressed vs. air-dried samples (Fig. 5a, b). Conversely, the phase (Supplementary Fig. 6) and amplitude signal (Fig. 5a, b) indicate that the magnitude of the piezoelectric response within domains is significantly different. For hot-pressed films, the amplitude response within domains is in excess of 100 pm (yellow-to-orange regions), with surrounding regions having values of ~60 pm (darker green) (Fig. 5a). The amplitude response from air dried is significantly lower with values of ~60 pm within domains (darker green) and surrounding regions < 40 pm (blue) (Fig. 5b). Domain sizes of ~100–200 nm are interpreted as piezoresponsive regions rather than single crystalline domains, and previous PFM studies show similar domain sizes in biological tissues^36^. In addition, Supplementary Fig. 6 shows the morphology and phase images directly corresponding to the amplitude images in Fig. 5. The piezoresponsive regions (brighter regions) in Fig. 5a, b are of similar size but do not clearly correlate to the position of globular structures of the surface topography, confirming there is no cross talk in the PFM measurements.

PFM switching spectroscopy (SS-PFM) measurements are further employed to elucidate the polarization switching dynamics of the differently treated cellulose films. Representative hysteresis loops of the hot-pressed sample (Fig. 5c) appear as a typical butterfly shape in the amplitude displacement (red curve) and the corresponding phase change is ≈ 180°, confirming a fully reversible polarization dynamic^37, 38^. The air-dried film, on the other hand, shows an unsaturated piezoresponse at positive biases (Fig. 5d), as indicated by significantly less amplitude displacement located on the right wing of the butterfly loop compared to the left wing or negative bias. In addition, the corresponding phase changes are far below 180°, indicating that a completely reversible polarization process is not achieved. This lower amplitude displacement and incomplete polarization switching is attributed to the lower-crystallinity degree though the shielding effect of residual water could also be a contributing factor in aired dried films. Generally, there are two types of water molecules in regenerated cellulose film, namely, the free water and bound water in the cellulose matrix^19^. Free water molecules are easily removed by heating treatment, however, the bound water can persist even at high temperatures. Statistical analysis of bias-induced piezoresponse at a maximum of −25 V shows the histogram peak distribution of the amplitude displacement for the hot-pressed film under 60 °C is threefold higher than the air dried (Fig. 5e), thus confirming differences observed in the PFM amplitude images. Similarly, the hot-pressed films under 100 °C show significantly higher amplitude displacement in the butterfly curves (Supplementary Fig. 7a), with peak distribution values of ~160 pm (Supplementary Fig. 7b) that are comparable to the 60 °C films. Therefore, a decrease in the water content accompanied by an increase in crystallinity gives rise to higher piezoelectric response and complete polarization in hot-pressed films.

## Discussion

In conclusion, we fabricate ME composites using cellulose as the active piezoelectric material, leading to considerable *α*_ME_ coefficients of 1.41 V cm^−1^ Oe^−1^. The cellulose and improvement of its crystallinity, piezoelectric properties, and consequently the ME output is easily achieved using simple and inexpensive solution processing methods. A Fano-like resonance, consisting of an anti-resonance dependence on the magnetic field strength, appears to be due to the unique chemical structure and properties of the cellulose. The asymmetric Fano-resonance profile demonstrates that it is possible to shift the ME output voltage from a peak value to zero sharply, enabling for accurate control of a relatively broad range of power output by easily manipulating the applied magnetic field, and further studies on the resonance line shape will be fundamental for understanding cellulose-based ME composites. The ME composites also exploit the current demand for cellulose as a renewable and cheap material, as well as biocompatible and biodegradable properties^39^ that, e.g., will progress the development of ME composites in a range of applications. In doing so, the study successfully demonstrates the concept of using naturally occurring piezoelectric biopolymers though we anticipate that other piezoelectric proteins and biological materials (e.g., collagen) will importantly find their way into ME composites.

## Methods

### Cellulose films preparation

Cellulose solution was prepared by using anhydrous dimethylacetamide (DMAc, Sigma-Aldrich 271012)/LiCl (Sigma-Aldrich 746460) solvent system and headed to 80 °C with constant stirring. The w.t. % of cellulose, DAMc, and LiCl are 1, 9, and 90%, respectively. To increase the solubility, α-cellulose (Sigma-Aldrich C8002) was first processed with pre-solution exchange method^40^. Briefly, the cellulose powder was suspended in DI water over night, and then moved to methanol for 1 h. After filtration, the cellulose was exchanged alternately in methanol and DMAc for four times. The result mixture of cellulose and anhydrous DMAc was stored under nitrogen protection before use. Cellulose films were fabricated through regular film casting process. Generally, 1.6 g of cellulose solution was spread on microscopy glass slide and the pre-film solution was evaporated in fume hood overnight. The casted gels were washed by a blended solution of DI water/isopropanol (3:1 in volume) and then immersed in DI water for 3 days with frequent water changing to remove the residual solvent. The cleaned gel-like films were dried in room temperature as control film, or clamped with hexamethyldisilazane-treated hydrophobic glass in vacuum oven at desired temperature to obtain hot-press film.

### ME laminate composite fabrication

The cellulose films were sputtering coated with 50 nm gold layers on both sides as interface electrodes and then were tailored into a 40 × 8 mm rectangular by using scalpel. To fabricate the ME laminate composites, a 36 × 6 mm Metglas 2605 SA1 plate was glued on the central part of cellulose films using commercial Devcon epoxy.

### Bulk ME effect measurement

An alternatingly generated Helmholtz coil was used to apply ac fields from 20.1 to 92.1 KHz and an electromagnet was use to conduct dc field with various strength. Both the ac and dc fields were provided along the length direction of the ME laminate composites. The induced output voltage was recorded by using a model SR8 10 DSP lock-in amplifier.

### Local piezoelectricity measurements

The local piezoelectric response of the cellulose films was analyzed by using an asylum MFP-3D atomic force microscopy system and the conductive tip was nanosensors EMF-50 (Pt/Ir coating) with resonant frequency of 68 kHz and spring constant of 2.8 N m^−1^. The morphology, amplitude, and phase images were obtained in dual ac response tracking^41^ with contact resonant frequency around 320 kHz. Piezoelectric response was measured as the first-harmonic of bias-induced tip deflection: *d* = *d*_0_ + *A*cos(*ωt* + *φ*), where *d*_0_ is the equilibrium position of the tip; *A* is the amplitude and *ω* is the frequency of applied bias; *φ* is the phase-yielded information on the polarization direction below the tip. To study the polarization-switching dynamic, the switching spectroscopy technique was used to obtain the local piezoelectric hysteresis loop. For each laminate sample, spectroscopy measurements were acquired across a 3 × 3 µm^2^ area by applying bias in a matrix of 7 × 8 points. The voltage during the SS-PFM measurement was applied in the range of ± 25 V, with the frequency of 1 Hz, and this loading is expected to switch the polarization component back and forth. We modified the system by connecting an external amplifier because the upper limit of output signal is ± 10 V, which is too weak to induce a saturated switching dynamic. The driving amplitude has been settled on 200 mV for all aforementioned measurements.

### DSC and TGA measurements

The thermograms of hot press and control cellulose films were obtained by using a TA Instrument Q100 DSC in the range of 25–450 °C with a heating rate of 10 °C min^−1^. TA Instrument SDT Q600 thermal analysis system was employed for thermogravimetric analyses. The measurements were run under an atmosphere with 9:1 oxygen/nitrogen flows from 25–425 °C at a ramping rate of 10 °C min^−1^.

### SEM

The cross-section morphology of air-dried and hot-pressed cellulose films were imaged by using SEM (JEOL JSM-7500FA) with the accelerating voltage of 5.0 kV and the emission current of 10 mA. The samples were sticked on a SEM specimen holder for cross-section imaging and sputter coated with 50 nm thick gold layer.

### Data availability

Data are available from authors on reasonable request.

## Electronic supplementary material

Supplementary Information
