# Supplementary material for: Cellulose-based magnetoelectric composites
Source: Nat Commun. 2017 Jun 28;8:38. doi: 10.1038/s41467-017-00034-4 (PMC5489539; doi:10.1038/s41467-017-00034-4)
Supplement: Supplementary file 1 — Supplementary Information [file 41467_2017_34_MOESM1_ESM.pdf]

File name: Supplementary Information
Description: Supplementary Figures and Tables

Supplementary Information

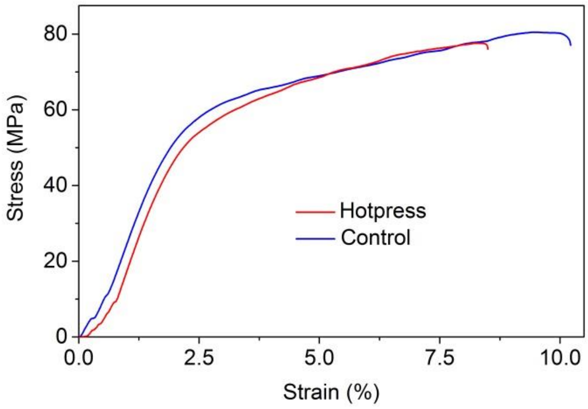

Supplementary Figure 1| Tensile tests of the aired control film and the hot pressed (60 ℃) film. The Young's modulus is calculated as 3.95 GPa for both two films**.**

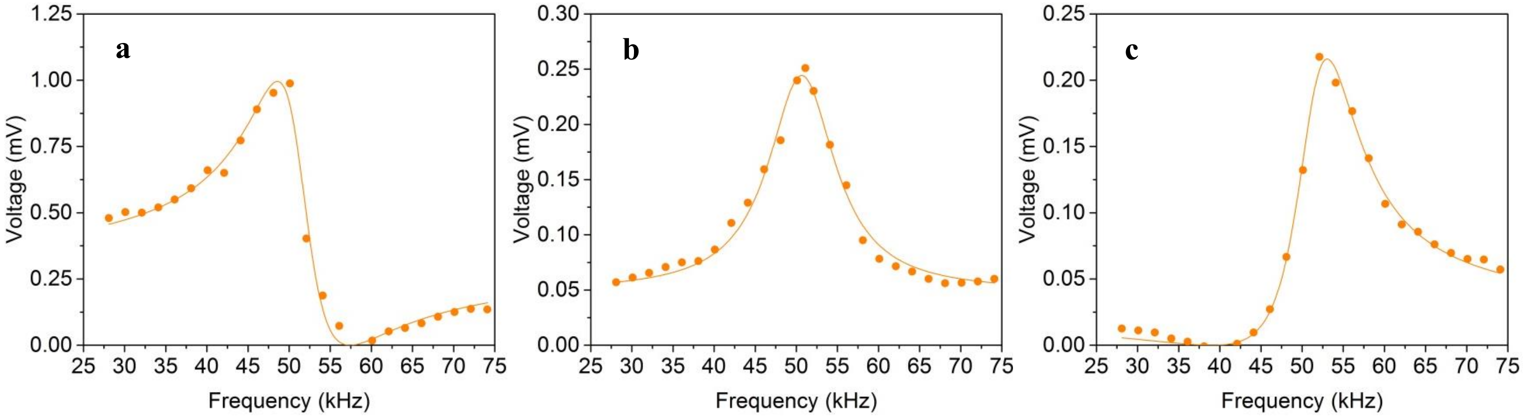

Supplementary Figure 2| ME output voltage of hot-pressed sample at 100 ℃ as a function of $\mathbf{H}_{ac}$ frequency under (a) $\mathbf{H}_{dc}$=4.2 Oe, (b) $\mathbf{H}_{dc}$=13.1 Oe and (c) $\mathbf{H}_{dc}$=19.0 Oe. The experimental data (dots) are fitted to a modified Maxwell equation (4) or Lorentzian function (shown as solid lines).

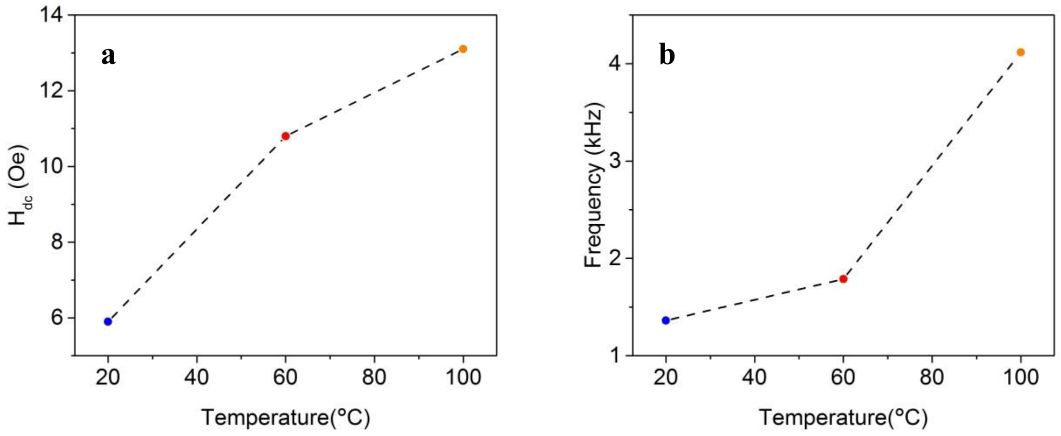

Supplementary Figure 3| The effect of water content on (a) the occurring $\mathbf{H_{dc}}$ strength of symmetric Lorentz resonance peak and (b) the frequency difference between the anti-resonance (f_a) and the resonance (f_r) peak under optimum $\mathbf{H_{dc}}$ strength. Note: the air dried sample is corresponding to 20 ℃ and the two hot-pressed samples are corresponding to 60 and 100 ℃. The f_a and f_r for the hot-pressed sample at 100 ℃ are 55.5183 and 51.0421 kHz, respectively. The f_a and f_r values for the other two samples can be found in supplementary tables 1 and 2.

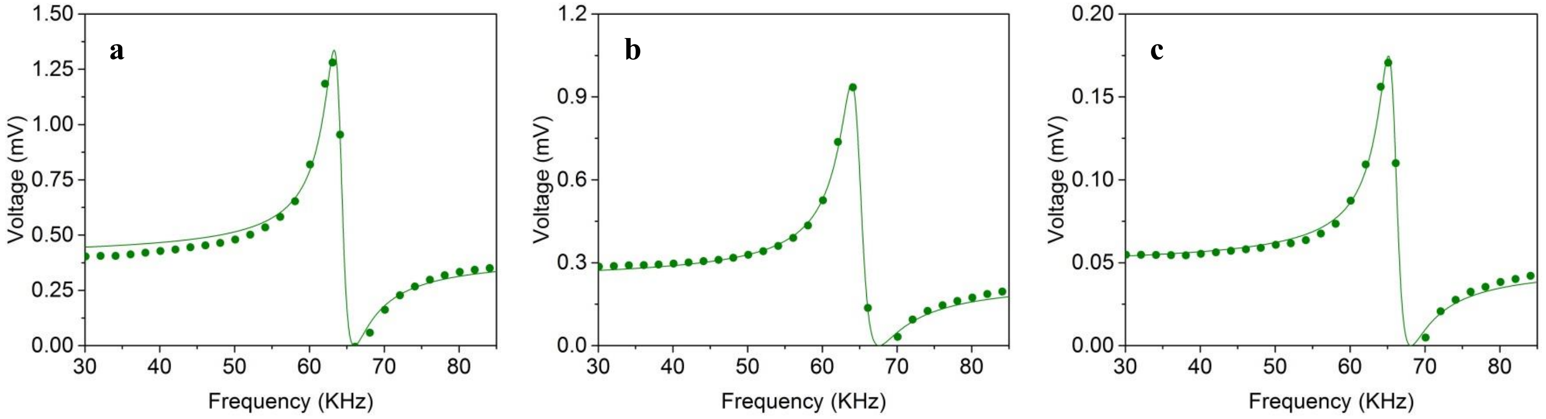

Supplementary Figure 4| ME output voltage of cellulose nanocrystal based ME laminate at as a function of $\mathbf{H_{ac}}$ frequency under (a) $\mathbf{H_{dc}}$=2.3 Oe, (b) $\mathbf{H_{dc}}$=12.2 Oe and (c) $\mathbf{H_{dc}}$=23.6 Oe. The experimental data (dots) are fitted to a modified Maxwell equation (4) or Lorentzian function (shown as solid lines).

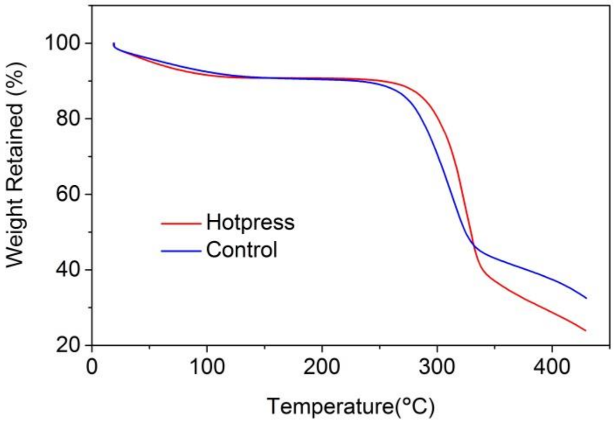

Supplementary Figure 5| TGA curves of hot-pressed (red line) and control (blue line) cellulose films, suggesting different decomposition temperature of the two films.

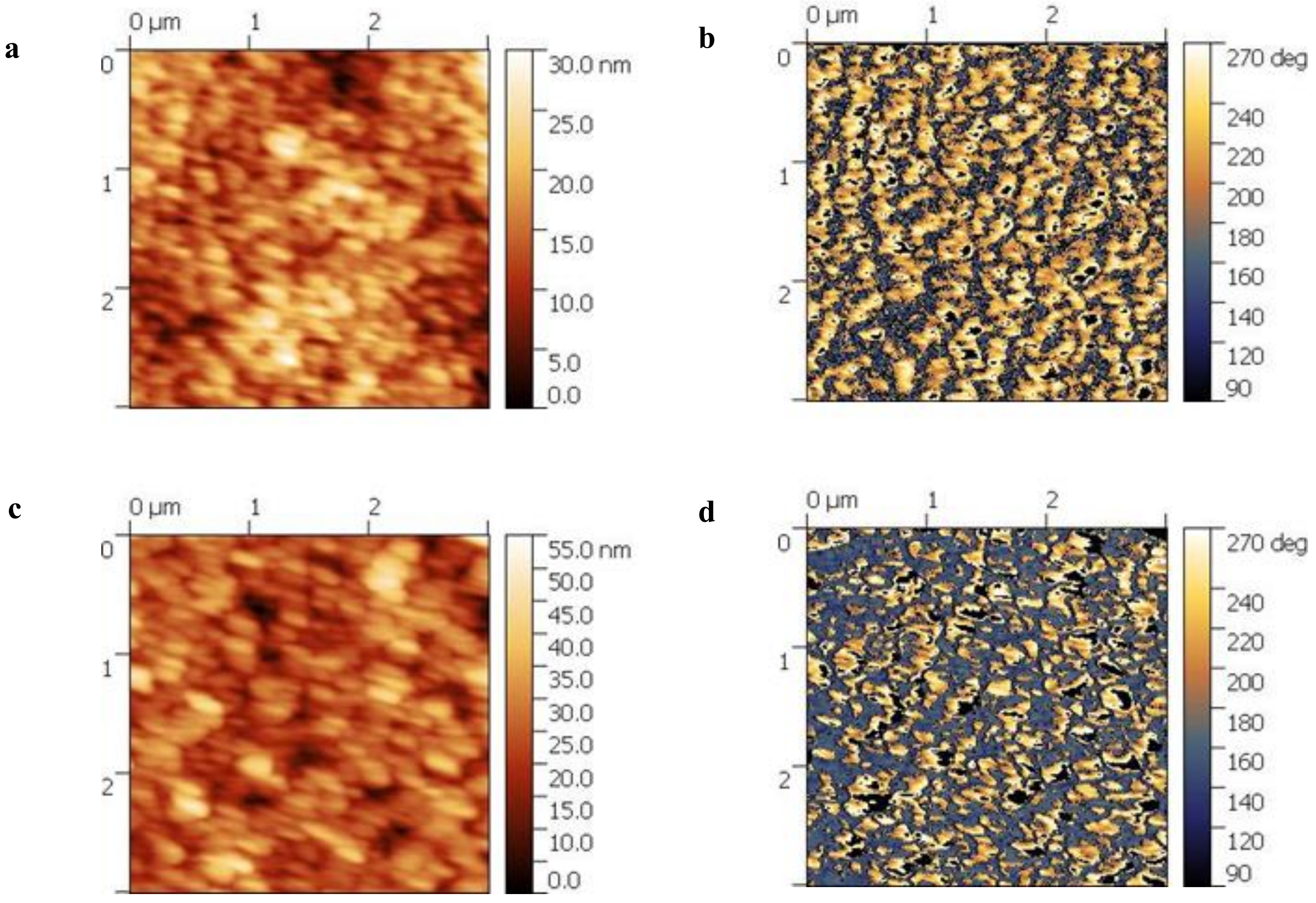

Supplementary Figure 6| PFM (a) height and (b) phase images of hot-pressed (60 ℃) cellulose film, and (c) height and (d) phase images of air dried cellulose film. A conductive tip was used to apply a constant amplitude of 9.4 V to induce local ME displacement while imaging.

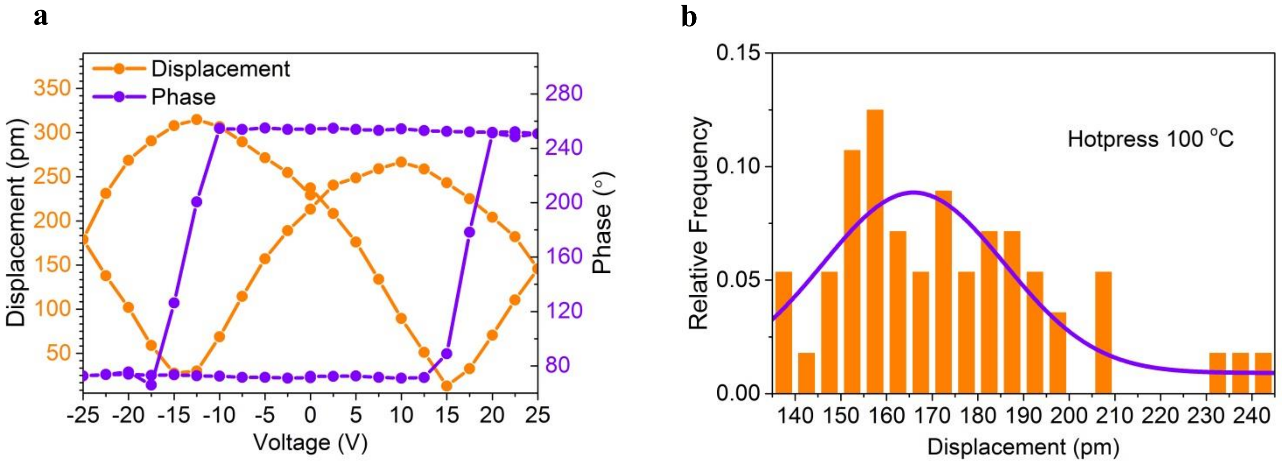

Supplementary Figure 7| (a) local piezoelectric response of hot-press cellulose film elucidated by using SS-PFM. The orange dots are the hysteresis loops representing the bias induce amplitude displacement. The violet dots represent phase changes corresponding to the hysteresis loops. (b) histograms of bias induced amplitude displacement at applied voltage of -25 V during SS-PFM measurements.

H_{dc} (Oe)	A (mV)	f_r (kHz)	f_a (kHz)	δ_r	δ_a
**2.2**	0.3881	54.8121	56.0363	-0.0240	0.0308
**4.2**	0.5125	55.4388	57.2276	-0.0246	0.0330
**7.1**	0.3653	55.7093	58.2671	-0.0257	0.0253
**12.8**	0.1077	55.6533	51.3223	0.0349	-0.0363
**15.8**	0.0655	56.1777	54.6733	0.0285	-0.0538
**19**	0.0512	56.7783	56.6211	0.0263	-0.0343
**28.8**	0.0049	57.3357	61.6959	0.0277	-0.1483

Supplementary Table 1| Fitting parameters (imaginary section) of equation (4) for anti-resonance frequency effect of hot-pressed cellulose ME laminate under different $\mathbf{H_{dc}}$ strength.

H_{dc} (Oe)	A (mV)	f_r (kHz)	f_a (kHz)	δ_r	δ_a
**1.9**	0.2039	55.5821	58.0353	-0.0336	0.0523
**3.9**	0.2102	55.8666	57.2276	-0.0312	0.0504
**7.6**	0.1414	55.6457	49.5998	0.0308	-0.0362
**10.2**	0.1027	56.2105	51.3223	0.0275	-0.0660
**12.7**	0.0682	56.4616	54.8179	0.0191	-0.0646
**20**	0.0512	56.2052	56.1057	0.0315	-0.0402
**29**	0.0232	58.1695	57.9086	0.0411	-0.0167

Supplementary Table 2| Fitting parameters (imaginary section) of equation (4) for anti-resonance frequency effect of air dried cellulose ME laminate under different $\mathbf{H_{dc}}$ strength.
